# ‘I Knew I Should Stop, but I Couldn’t Control Myself’: a qualitative study to explore the factors influencing adolescents’ consumption of sugar-sweetened beverages and sugary snacks from a socio-ecological perspective

**DOI:** 10.1017/S1368980022001185

**Published:** 2022-09

**Authors:** Chia-Wen Wang, Duan-Rung Chen, Chang-Chuan Chan, Yen-Po Yeh, Hsiu-Hsi Chen

**Affiliations:** 1 Innovation and Policy Centre for Population Health and Sustainable Environment (Population Health Research Centre, PHRC), College of Public Health, National Taiwan University, Taipei, Taiwan; 2 Institute of Environmental and Occupational Health Sciences, College of Public Health, National Taiwan University, Taipei, Taiwan; 3 Institute of Health Behaviours and Community Sciences, College of Public Health, National Taiwan University, No. 17, Xu-Zhou Rd., Taipei 100, Taiwan; 4 Changhua County Public Health Bureau, Changhua City, Changhua County, Taiwan; 5 Institute of Epidemiology and Preventive Medicine, College of Public Health, National Taiwan University, Taipei, Taiwan

**Keywords:** Sugar-sweetened beverages, Sugary snacks, Qualitative study, Adolescents, Added sugar

## Abstract

**Objective:**

To explore the factors influencing Taiwanese adolescents’ consumption of sugar-sweetened beverages (SSB) and sugary snacks from a socio-ecological perspective.

**Design:**

This study adopted a qualitative design by using face-to-face, in-depth interviews guided by a semistructured questionnaire.

**Setting:**

Eight junior high schools in New Taipei City and Changhua County, Taiwan, September to November 2018.

**Participants:**

Fifty-nine participants aged 12–14 years participated in this study.

**Results:**

Reflexive thematic analysis was used to analyse the data. This study identified four themes to address the multifaceted factors that influence adolescents’ consumption of SSB and sugary snacks. At the intrapersonal level, physiological factors, psychological factors, individual economic factors and taste preferences were mentioned in connection with people’s consumption of SSB and sugary snacks. Positive or negative influences of parents, siblings, peers and teachers on SSB and sugary snack intake were identified at the interpersonal level. The availability of SSB and sugary snacks at home, their availability in vending machines or in school stores in the school environment and participants’ access to convenience stores and hand-shaken drink shops in the broader community influenced SSB and sugary snack consumption. Additionally, food culture and food advertising were identified as influencing societal factors.

**Conclusions:**

Overall, this qualitative study determined not only that the consumption of SSB and sugary snacks is influenced by intrapersonal factors but also that interpersonal, environmental and societal factors affect adolescents’ increased sugar intake. The findings are helpful to broaden the options for designing and developing interventions to decrease SSB and sugary snack consumption by adolescents.

Over the last four decades, the number of children and adolescents with obesity has dramatically increased worldwide, from 11 million in 1975 to 124 million in 2016^(1)^. Overweight and obesity in children and adolescents are associated with adverse health consequences, such as early-onset diabetes^(2)^, sleep problems^(3)^ and depression^(4)^. Increased intake of added sugar, particularly from sugar-sweetened beverages (SSB) and sugary snacks, is a major health concern related to an increased risk of obesity in children and adolescents^(5)^. Hence, the WHO suggests that free sugar intake should be less than 10 % of the total energy intake to reduce the risk of non-communicable diseases in children and adults^(6)^.

Globally, the regions with the highest SSB consumption are the Caribbean, central Latin America and North America; however, the young generation in Taiwan has the highest daily intake of SSB in East Asian countries^(7)^. Taiwan, located in East Asia, has a unique food culture and food environment, and eating out is a common dietary habit. There are a wide variety of available options for eating out. The ubiquitous breakfast shops, convenience stores and street food vendors contribute to the overconsumption of daily added sugar among Taiwanese people.

Well-known hand-shaken drink shops are widespread throughout Taiwan, with more than 20 000 such shops countrywide^(8)^. Hand-shaken beverages^(9)^ are drinks consisting of tea, tapioca (or other ingredients, such as jelly, pudding or fruits), ice and added sugar, all shaken together. In North American and European countries, frequently consumed SSB are regular sodas, sports drinks, energy drinks, fruit drinks and coffee and tea beverages with added sugar^(10)^. However, hand-shaken beverages are a typical source of daily SSB intake in Taiwan because they represent an important local food tradition (boba). Given the convenience and accessibility of SSB and sugary snacks in Taiwan, adolescents have increased their intake of added sugar, posing an alarming threat to their well-being. The national nutrition and health survey reported that 49·0 % of Taiwanese adolescents aged 13–15 consumed SSB daily. Furthermore, 10·8 % of these adolescents had overweight, and 13·5 % had obesity^(11)^.

Adolescents today experience challenges in adopting healthy eating habits because food systems need to be transformed to support such habits^(12,13)^. Adolescents are particularly vulnerable to consuming energy-dense, low-nutrient foods and beverages due to complex determinants. The socio-ecological approach^(12,14)^ offers a comprehensive conceptual framework to understand the multifaceted factors that influence unhealthy diets. SSB and sugary snack consumption are determined by intrapersonal-level influences such as nutritional knowledge^(15)^, self-efficacy^(16)^ and taste and preference^(17)^. Additionally, interpersonal factors (e.g. parental influences^(17,18)^ and peer-related influences^(17,19,20)^), environmental influences^(17,18,21,22)^ and macrolevel and policy environments^(23,24)^ are key drivers of SSB and sugary snack intake. Therefore, multifaceted strategies are needed to reduce added sugar intake in adolescents^(25)^. In this vein, interventions at the intrapersonal level (e.g. improving nutritional knowledge^(12)^ and self-efficacy) and interpersonal level (e.g. education campaigns tailored to caregivers or peers), a food environment that supports health (e.g. providing free, tasty and healthy school meals^(12)^), and food policies (e.g. a sugar tax^(23)^ and restriction of the sale of SSB^(24)^ on school grounds) are all crucial for supporting reduced SSB and sugary snack intake by adolescents in daily life.

There is a lack of studies that explore the multifaceted influences on SSB and sugary snack consumption to broaden the understanding of this phenomenon in adolescents. Therefore, this qualitative study aimed to explore adolescents’ perspectives and experiences with respect to SSB and sugary snack consumption from a socio-ecological perspective to inform effective multifaceted interventions.

## Methods

### Study design, participants and setting

This qualitative study was conducted from September to November 2018 in New Taipei City and Changhua County in Taiwan using a convenience sampling method. New Taipei City and Changhua County were selected as typical urban and rural settings, respectively (based on the cropland land rate in these two places, 11·90 % and 56·97 %^(26)^, respectively). Two schools in New Taipei City were included in this study through the social contacts of members of the research team, and six schools in Changhua County were included in this study through the Changhua County Public Health Bureau. In total, eight schools agreed to participate in our research. The target participants were students in grades 7–9 who were 12–14 years old. The schools varied in size, ranging from as few as seventy-five students in rural areas to as many as 1799 students in urban areas. Two schools in rural areas had vending machines that sold beverages as well as sugary snacks.

Additionally, two schools in rural areas and two schools in urban areas had one store. Each school was equipped with water dispensers (machines for cooling/heating and providing drinking water); the number of water dispensers in each school ranged from as few as 6 (rural areas) to 61 (urban areas). The characteristics of the eight schools included in this study are described in the online supplementary material (see online supplementary material, Supplemental Table 1).

### Procedure and ethical considerations

Teachers announced the relevant research information and interview opportunities in their classrooms to recruit student volunteers. Subsequently, students who wanted to volunteer contacted their teachers to express their willingness to participate in our study. The volunteer students were given informed consent forms to take home to have enough time to discuss the study with their parents or legal guardians. Written consent forms were signed by both the students and their parents or legal guardians. After the informed consent process, we arranged an appropriate location and time to interview the students. We provided coupons valued at NT 300 (equivalent to approximately 10·6 US dollars in market exchange rate at the time of writing) as well as snacks or lunch boxes as incentives for participation in the face-to-face, in-depth interviews.

### Data collection

Data were collected through face-to-face, in-depth interviews guided by a semistructured questionnaire. An interview guide was developed to investigate various factors influencing SSB and sugary snack consumption, including intrapersonal, interpersonal, environmental and societal factors, through a socio-ecological lens. A guide was developed to direct the interviews (see online supplementary material, Supplemental Table 2). The interviews were conducted by two research assistants who were trained by D.R.C. before the interviews to minimise potential bias and ensure the research quality. The interviews were conducted in Chinese in a designated room (occupied only by one research assistant and one student) in each school, and each interview lasted 30 to 60 min. After the interviews, demographic information, such as age, sex, weight and height, was collected by administering a short-structured questionnaire. We recruited the participants until data saturation was achieved (no new data and enough information for this study).

### Data analysis

All the interviews were recorded, and the audio recordings were transcribed verbatim in Chinese, including hesitations, laughter and long pauses. Reflexive thematic analysis^(27,28)^ was used to analyse the data. The data analysis started with familiarisation, whereby two researchers (C.W. W. and D.R.C.) read and reread all the transcripts of the interviews and recorded notes and comments until they were thoroughly familiar with the data. All transcripts were imported into NVivo 12 software (QSR International, Melbourne, Australia) for analysis. C.W.W. performed the following coding process. Initial coding incorporating *in vivo* coding was used for the first cycle of the coding process^(29)^. Each transcript was entirely coded before moving to another. After the first codes were generated, they were used to code the relevant data. During the coding process, new codes were developed to capture the meaning of data, and existing codes were modified to integrate new material^(28)^. The process was repeated throughout all the transcripts. Pattern coding was used in the second cycle coding process to group similar codes into categories^(29)^. These categories were identified based on the different levels of influence on SSB and sugary snack consumption through a socio-ecological analytic lens. Based on the coding analysis, categories were further refined, and themes were generated. To improve the credibility of the data analysis, D.R.C. and C.W.W. discussed and refined the codes, categories and themes until consensus was achieved. All analyses were conducted in Chinese to maintain the intended meaning. For publication purposes, the quotes, codes and categories were translated from Chinese into English by a bilingual researcher to ensure accurate representation.

### Researcher characteristics and reflexivity

The five researchers have doctoral degrees in public health or sociology. C.W.W. (female), D.R.C. (female), C.C.C. (male) and H.H.C (male) work in academia in public health. Y.P.Y. (male) is the director of the Changhua County Public Health Bureau and has extensive experience promoting public health in the community. Our backgrounds in public health and/or related work experiences in the community may have an impact on the collection, analysis and interpretation of the data.

## Results

In total, fifty-nine participants aged 12–14 years were interviewed in this study. Of the fifty-nine participants, 50·8 % lived in New Taipei City and 49·2 % lived in Changhua County. The average height and weight of the participants were 159·5 (±7·5 sd) cm and 52·8 (±13·2 sd) kg, respectively. Of the fifty-nine participants, 25·4 % were overweight or obese (Table 1).

**Table 1:** Characteristics of the participants (*n* 59)

	Male (*n* 27)	%	Female (*n* 32)	%	Total (*n* 59)	%
Age						
Mean	13·2		13·3		13·3	
sd	0·7		0·6		0·7	
12	5	18·5	2	6·3	7	11·9
13	12	44·4	17	53·1	29	49·2
14	9	33·3	13	40·6	22	37·3
Missing	1	3·7	0	0·0	1	1·7
Residence						
New Taipei City	16	59·3	14	43·8	30	50·8
Changhua County	11	40·7	18	56·3	29	49·2
Height/weight						
Height (cm)						
Mean	161·5		157·8		159·5	
sd	9·5		4·7		7·5	
Weight (kg)						
Mean	51·8		53·5		52·8	
sd	11·1		14·8		13·2	
Missing	1		0			
BMI (kg/m^2^)*						
Mean	19·7		21·5		20·7	
sd	3·6		5·7		4·9	
Underweight	3	11·1	0	0·0	3	5·1
Normal	18	66·7	22	68·8	40	67·8
Overweight	1	3·7	4	12·5	5	8·5
Obese	4	14·8	6	18·8	10	16·9
Missing	1	3·7	0	0·0	1	1·7

The total percentages may differ from 100 due to rounding.

* The BMI-for-age cut-offs recommended by the Health Promotion Administrations, Ministry of Health and Welfare, Taiwan, were used to assess overweight and obesity.

Most participants (93·3 %) reported drinking SSB at least once per week, and 32·3 % consumed SSB every day. Regarding the types of SSB, participants reported drinking breakfast shop beverages (black tea, milk tea or soy milk with added sugar), hand-shaken drinks and packaged beverages, such as beverages in cartons or bottles. In addition, 54·2 % of the participants reported eating sugary snacks at least once per week. The sugary snacks mentioned included chocolates, popsicles, potato chips, shaved ice, cakes, tofu pudding, flavoured gelatine, candy and cookies. This study identified four themes, seventeen categories and thirty-eight codes (see online supplementary material, Supplemental Table 3) to address the multifaceted factors that influence SSB and sugary snack consumption by adolescents. These themes, categories and codes are presented with supporting quotes below.

### Theme 1: Intrapersonal factors influence SSB and sugary snack consumption

#### Physiological factors

Quenching thirst and reducing hunger were mentioned by participants as reasons for their consumption of SSB and sugary snacks. In particular, participants described feeling thirsty after exercise and perceiving a physical need to replenish sugar as reasons to drink SSB. In contrast, some participants used sugary snacks to satisfy hunger between regular meals. Reducing sleepiness was also a reason that was frequently mentioned. Participants noted that SSB and sugary snacks could reduce fatigue and improve their concentration.

*‘I think eating chocolate can refresh me. I eat chocolate while I am studying. By doing that, I feel I can concentrate more on studying.’ [14, F]*

#### Psychological factors

Negative psychological states were linked to SSB and sugary snack consumption. Participants stated that they consumed SSB and sugary snacks when they felt bad, angry, stressed, bored or tired. SSB and sugary snack consumption appeared to be a means by which to improve their mental status. In addition, the participants perceived motivation as one of the psychological benefits of SSB and sugary snack intake.

*‘There are psychological benefits of them (SSBs). I feel refreshed and happy after drinking them. After that, I feel much more motivated to do something that I would like to do.’ [14, M]*

Furthermore, the participants stated that they or their schoolmates seemed to be addicted to SSB and sugary snacks. They knew that these foods were bad for their health, yet they could not stop drinking or eating them.

*‘I kept eating and eating (potato chips), and I felt that I couldn’t stop it. I knew I should stop, but I couldn’t control myself. Then, I ate one more pack of potato chips after eating one pack, and then I took another. I couldn’t stop eating.’ [12, F]*

*‘If you get used to drinking them (SSBs), you will probably get addicted to them. Like an older student I know, he drinks bubble tea every day, and he says he has become addicted to it. I feel afraid of that.’ [14, F]*

#### Individual economic factors

Participants stated that their purchases of SSB and sugary snacks depended on the allowance their parents provided. In addition, the prices of SSB and sugary snacks affected participants’ purchase of these foods. Some participants mentioned that MineShine, one of the regularly consumed SSB in cartons, was easily affordable for them.

*‘I got NT 150 in allowance every day. I used it to buy my dinner and sweetened white gourd drink.’ [13, F]*

*‘My mom gives me a limited allowance so that I will save money on my own. Then, I can use my savings to buy beverages…it is painful to use my savings to buy beverages, so I chose MineShine (a local beverage brand in Taiwan) green tea because it only cost NT 10.’ [12, F]*

*‘If I have money on hand and I feel thirsty, then I will buy the cheaper and bigger one (SSB).’ [12, M]*

#### Taste preferences

Taste also seemed to be one of the participants’ reasons for consuming SSB and sugary snacks. The participants noted that SSB and sugary snacks taste sweet or good. When asked what they chose when water and SSB were available, participants reported selecting SSB since SSB taste better than water.

*‘I felt happy because it (an SSB) tasted sweet. It tasted good because it was sweet.’ [14, F]*

*‘I like drinking water…But I will choose to drink sweet beverages if they are available. They taste good and sweet.’ [14, F]*

### Theme 2: Interpersonal factors influence SSB and sugary snack consumption

Participants reported various positive and negative influences of their parents, siblings, peers and teachers that affected SSB and sugary intake at the interpersonal level.

#### Negative parental influences

A few participants reported that their parents regularly consumed SSB and sugary snacks at home and that they consumed these foods together when they saw their parents doing so. Lack of parental supervision also appeared to influence participants’ SSB or sugary snack consumption. Finally, participants reported that their parents provided SSB and sugary snacks.

*‘They (parents) say nothing (if they see me drink SSBs or eat sugary snacks). Instead, they buy hand-shaken drinks and sugary snacks for me sometimes.’ [14, F]*

#### Positive parental influences

Parents can be critical positive role models regarding SSB and sugary snack consumption for their children. The majority of the participants noted that their parents consumed fewer SSB and sugary snacks than they did. In addition, participants said that parental supervision and control influenced their consumption of SSB and sugary snacks. Participants told their parents to admonish or stop them when they were consuming too many of these items.

*‘My mom would say, “Do not drink too much (beverage).” She recommends that I drink beverages with no sugar; however, I feel they taste bitter with no sugar, so I usually drink beverages with low sugar.’ [14, M]*

#### Negative sibling influences

In addition to their parents’ influences, participants mentioned that sibling influences affected their consumption of SSB. For example, they went with their siblings to buy SSB and sugary snacks. The participants also described food-sharing behaviour between siblings.

*‘My sister convinces me to go together to buy beverages. However, she always takes one or two sips, and then she gives me the rest of the drink.’ [12, F]*

#### Negative peer influences

Peers have a significant influence on SSB and sugary snack intake by adolescents. In the interviews, participants expressed the perception that their peers regularly consumed these foods at school. In addition, food sharing behaviour among peers, food recommendations from peers and peer pressure were social forces that prompted participants to consume SSB and sugary snacks.

*‘I went out with my classmates. We went to the hand-shaken drink shop, and they all bought hand-shaken drinks. On that occasion, I felt that I had to buy a beverage with them; it would be very weird if I were the only one who didn’t do that.’ [13, F]*

#### Negative teacher influences

In the interviews, participants mentioned that when their teachers consumed SSB at school, they also wanted to drink SSB. Lack of supervision from the teachers also appeared to influence participants’ SSB or sugary snack consumption. In some instances, SSB and sugary snacks were used by teachers as an incentive or reward to encourage students to perform well.

*‘He/she (the teacher) pretended that he/she didn’t see us drink the beverages.’ [13, F]*

*‘For example, if we got good scores on a test, the teachers would reward us, and they would buy ice cream or beverages to treat us.’ [14, F]*

#### Positive teacher influences

Teachers were also crucial positive role models for adolescents’ eating behaviours. For example, teachers who drank beverages with no sugar or water at school were healthy beverage role models for participants.

*‘I always see him (the teacher) drink water…Also, I don’t see him eat sugary snacks.’ [13, M]*

In addition, participants mentioned that some of the teachers supervised or regulated their consumption of SSB or sugary snacks. Participants from rural areas reported that teachers assigned them exercises as punishment if they violated these rules.

*‘Our teacher will check whether you bring them (SSBs) into the school. If you do, you will be punished and have to do push-ups.’ [13, F]*

### Theme 3: SSB and sugary snack consumption are influenced by the environmental context

#### Food environment at home

Parents are gatekeepers of SSB and sugary snacks at home, and the availability of these items at home might prompt their children to consume them.

*‘My parents buy a lot of beverages at one time and then store the beverages in the fridge. If you want to drink one, you can take it yourself. They (my parents) buy one box of black tea or the green tea Yakult.’ [14, M]*

#### Food environment at school

The school setting is important for adolescents since they spend a substantial amount of time studying at school. Water dispensers were accessible in the eight schools located in rural and urban settings (see online supplementary material, Supplemental Table 1). In addition, the field investigation and interviews revealed that two of the rural schools had vending machines that sold beverages and sugary snacks, and four schools (rural and urban) had stores in the school. Participants reported purchasing SSB and sugary snacks from the stores and vending machines found in the schools.

*‘Many of my classmates eat candy, cookies, or crackers. They buy crackers from the vending machine because it sells very yummy crackers, such as cheese- or chicken-flavoured crackers.’ [13, M]*

*‘My classmates drink beverages daily. They drink one cup in the morning, and they go to the store (at school) to buy beverages (juice or black tea).’ [14, F]*

#### Food environment in the neighbourhood near home

In general, the majority of the participants reported that it was convenient to purchase SSB and sugary snacks in the neighbourhood near their home.

*‘There are many shops and stores that sell beverages near my home. For example, if I go downstairs, there is a supermarket on the first floor of my home, and if I go out of the neighbourhood, there are three convenience stores, such as 7–11 and Family Mart, and hand-shaken drinks shops are nearby.’ [12, F]*

However, a few participants from rural settings stated that there were few or no convenience stores or hand-shaken drink shops in the neighbourhoods near their homes; therefore, it was inconvenient for them to buy or obtain SSB or sugary snacks.

*‘There are only farms surrounding my home; there are no beverage shops nearby. If I would like to buy beverages, I have to ride a bicycle to the school nearby to get it.’ [13, F]*

*‘I live in Shuiwei (a village located in Changhua County), and there is only one grocery store nearby. It is a rural area, so there are no convenience stores. It takes five or ten minutes by car to arrive in an urban area.’ [13, F]*

#### Food environment in the neighbourhood near school

The food environment in the neighbourhood near school is an important determinant of adolescents’ eating behaviour, particularly in Taiwan. Taiwanese adolescents usually buy breakfast with SSB at breakfast shops in the neighbourhood near school before entering school. After school, they buy or eat dinner with SSB in the neighbourhood near school before entering their cram schools (in Taiwan, some students attend extracurricular academic lessons after school to pass the high school entrance exam). Overall, participants reported that it was easy and convenient to buy or obtain SSB and sugary snacks in the areas around their schools. The food environment in each neighbourhood near each school is described in detail in Supplemental Table 1.

*‘It is easy to get them (SSBs) because there is a hand-shaken drink shop near our school gate. When I go to my cram school and pass by it, I buy a beverage.’ [13, M]*

#### School food policy

On a large scale, school food policy also has an impact on adolescents’ SSB consumption. There is no specific SSB policy in urban settings. However, the rural setting of Changhua County imposed a regulation banning SSB on school campuses in 2017. This regulation limits some types of SSB, including but not limited to hand-shaken drinks, sports drinks, apple-flavoured milk, cola and non-100 % fruit juice. However, some SSB can be sold in school if they have fewer than 250 calories and have < 30 % added sugar. For instance, beverages such as 100 % fruit juice, buttermilk and soy milk are allowed.

*‘There is no milk tea and black tea at the store. It sells beverages like soy milk and fruit juice.’ [13, F]*

### Theme 4: Societal-level characteristics influence SSB and sugary snack consumption

#### Food culture

Food culture has a significant influence on diet and eating behaviours. In Taiwan, hand-shaken drinks and breakfast are two crucial components of daily life. In the interviews, participants mentioned a popular type of SSB from hand-shaken drink shops, namely, bubble tea. Hand-shaken drinks are commonly consumed beverages in Taiwan.

*‘I usually go to 50 Lan (a local hand-shaken drink shop in Taiwan) to buy bubble tea with half sugar and no ice.’ [14, F]*

In addition, participants reported that they bought SSB (black tea, milk tea or soy milk with sugar) when they purchased breakfast at breakfast shops or convenience stores and took these foods to school to eat. Taiwan is well known for its breakfast culture, with a variety of dining options on the street. It is common to drink SSB with breakfast in Taiwanese society.

*‘I usually buy my breakfast at breakfast shops and drink them (SSBs) with my breakfast (sandwich or egg pancake roll) … It is black tea or soy milk with sugar (500 cubic centimetres).’ [12, M]*

#### Food advertising

Food advertising may affect the types of foods that adolescents consume. Participants reported that advertising made them want to consume SSB or sugary snacks. The advertising avenues included television, store and shop flyers, YouTube channels and social media, such as Facebook or Instagram.

*‘Advertisements for sugary snacks are shown on TV or the internet, and they recommend some delicious sugary snacks. If the sugary snack looks cool and looks like it will taste good, then I will go buy it.’ [12, M]*

## Discussion

This qualitative study generated some of the first evidence of the multifaceted factors influencing the consumption of SSB and sugary snacks by adolescents from a socio-ecological perspective to highlight intrapersonal, interpersonal, environmental and societal influences. This study also provides an understanding of adolescents’ SSB and sugary snack consumption with complex determinants to inform and develop practical multilevel approaches to reduce such intake.

At the intrapersonal level, the results suggested that thirst and hunger were common reasons that students consumed SSB and sugary snacks. Educational interventions can involve programmes such as campaigns about drinking water^(30)^ or maintaining healthy diets^(31)^ to encourage students to drink water or eat healthy snacks when they feel thirsty or hungry. Consistent with previous studies^(32,33)^, the results showed that adolescents’ mental states affected their consumption of SSB and sugary snacks. Individual interventions such as healthy stress or emotion management training by schools can enhance students’ ability to avoid SSB and sugary snacks when they experience a negative psychological state. In addition, the price of SSB and sugary snacks and the amount of students’ allowance could affect their consumption of SSB and sugary snacks. The findings suggested that food prices were considered when adolescents bought SSB and sugary snacks. A previous study demonstrated that excise taxes on SSB to increase SSB retail prices could reduce SSB purchases and consumption^(34)^. Legislative interventions can introduce taxes on sugary foods to encourage adolescents to choose cheaper and healthier foods.

At the interpersonal level, this study found that adolescents’ families, peers and teachers influence their consumption of SSB and sugary snacks. These people are critical role models for the development of healthy eating behaviour in adolescents throughout their lives. The findings suggested that parental SSB and sugary snack consumption, as well as a lack of parental supervision, can influence adolescents’ SSB and sugary snack intake, which is consistent with previous studies^(35,36)^. In addition, the home environment is important in determining the availability of SSB and sugary snacks to adolescents^(18,37,38)^. Thus, effective interventions tailored to parents include training parents to serve as healthy dietary role models, increasing the availability of healthy snack food and reducing the availability of SSB and sugary snacks at home. The influence of peers on the consumption of SSB and sugary snacks has been investigated in previous studies on topics such as the peer environment^(35)^, peer pressure^(17)^, peer norms^(20)^ and friendship group consumption^(19)^. Consistent with these studies, this study found that peers influenced adolescents’ SSB and sugary snack consumption. Interventions based on peer influence, such as social network-based interventions^(39)^ or peer-led education programmes^(40)^, can decrease adolescents’ SSB and sugary snack consumption.

The findings indicated that teachers’ consumption of SSB and sugary snacks might impact adolescents. Additionally, some teachers may use SSB and sugary snacks as incentives/rewards to encourage adolescents to perform well. Interventions tailored to teachers can encourage teachers to frequently drink water in front of their students to demonstrate healthy beverage habits^(41)^ or use nutritious food to reward students.

The results showed that the school food environment, including vending machines and school stores, also increased the availability of SSB and sugary snacks, contributing to higher consumption by students. These results are consistent with those of prior research^(21,42)^. School food environment programmes could, for instance, remove SSB and sugary snacks from vending machines and school stores and provide free or subsidised fruits or vegetables for students as strategies to reduce SSB and sugary snack intake. In the present study, although the students had access to water dispensers at school (see online supplementary material, Supplemental Table 1), the numbers of water dispensers at a few schools were inadequate considering the total numbers of students at these schools. For example, approximately 50 students shared one water dispenser in two schools (schools 3 and 7). Thus, some students might not have enough time to visit the water dispenser during their 10-minute class breaks. Increasing access to water by installing more water dispensers in schools may help to encourage students to drink water instead of SSB. Additionally, schools have an important responsibility to ensure the availability of safe and clean drinking water through the regular maintenance and inspection of water dispensers in schools.

Furthermore, this study found that the food environments of the neighbourhoods near school and home affected SSB and sugary snack consumption. In the present study, there were no notable differences in adolescents’ SSB or sugary snack consumption between New Taipei City and Changhua County. Overall, except for a few students who lived in highly rural communities, adolescents could easily buy SSB or sugary snacks in the neighbourhoods near their schools and homes. This study found that in both rural and urban settings, the school food environment includes many fast-food restaurants, hand-shaken drink shops, convenience stores and breakfast shops (see online supplementary material, Supplemental Table 1). It is easy and convenient for students to access SSB and sugary snacks, which increases their daily added sugar intake. Previous studies have shown that greater neighbourhood access to convenience stores^(43–45)^, fast-food restaurants^(46)^ and combination grocery and other stores^(47)^ is associated with an alarming increase in the risk of obesity in adolescents. A supportive neighbourhood food environment is essential for successfully reducing sugar intake in adolescents. The school neighbourhood food environment could be improved by implementing related public policy. For example, the government should increase business taxes on hand-shaken drink shops, convenience stores and fast-food restaurants built within 500 m of schools. Additionally, breakfast shops could receive tax reductions if they provide healthy menus and beverages with no added sugar to students.

Concerning food culture, breakfast and hand-shaken drinks are considered critical to the daily diets of the general population in Taiwan. Although Changhua County (rural setting) has implemented regulations that ban some SSB on campus, the results showed that SSB were brought into schools by students for consumption with breakfast. Interventions targeting the school food environment can provide free healthy breakfasts for students, facilitating the consumption of nutritious breakfasts instead of breakfasts purchased from outside breakfast shops. Legislative interventions can introduce a sugar tax on SSB and sugary snacks to reduce the intake of these products. Many countries, such as the UK, Finland, the UAE, Mexico and Thailand, have introduced a sugar tax on foods and drinks^(48)^. However, there is a lack of governmental food and tax policies on SSB and sugary foods in Taiwan. Evidence has suggested that SSB taxation is effective in reducing SSB consumption^(49)^. Furthermore, this study found that exposure to food advertising may contribute to SSB and sugary snack consumption. Because adolescents are vulnerable to overconsumption of added sugar, the government should introduce a tax system for SSB and unhealthy foods and related public policy to ban advertisements for sugary foods and facilitate the building of a healthy community for the next generation.

In general, this qualitative study suggests that SSB and sugary snack consumption are influenced by multifaceted factors from the intrapersonal level to the societal level. The evidence indicates that SSB interventions targeting individuals, the environment or both can effectively decrease SSB consumption; legislative/environmental interventions have had a 90 % success rate^(50)^. Because hand-shaken drink shops, breakfast shops and convenience stores are ubiquitous in Taiwan, legislative/environmental interventions may effectively decrease SSB and sugary intake in adolescents.

Although this qualitative study contributes a deeper understanding of the factors that influence SSB and sugary snack consumption in the Taiwanese sociocultural context, it has some potential limitations. First, it relied on voluntary participants, and self-selection bias could not be avoided. Therefore, the results might not sufficiently represent the grade 7–9 students in each school. Second, the findings from this study may be biased due to the small sample size. Hence, they are not necessarily representative of all Taiwanese junior high school students. Third, due to the private nature of the topic of dietary intake, this study may have been affected by social desirability bias and failed to capture some critical aspects across intrapersonal, interpersonal, environmental and societal influences. Fourth, although a socio-ecological model was used in this qualitative study, it was challenging to identify the effects on SSB and sugary snack consumption interactions across levels. Thus, crucial interactions across levels may be omitted when designing effective packages of interventions.

## Conclusion

Overall, this qualitative study determined that intrapersonal factors influence the consumption of SSB and sugary snacks and that interpersonal, environmental and societal factors also facilitate adolescents’ increased sugar intake. The findings can help broaden options for designing and developing interventions to decrease adolescents’ SSB and sugary snack consumption. These findings can have benefits beyond Taiwan as many adolescents worldwide develop unhealthy diets.

## Supplementary material

For supplementary material accompanying this paper visit https://doi.org/10.1017/S1368980022001185. click here to view supplementary material
