# Supplementary material for: ‘I Knew I Should Stop, but I Couldn’t Control Myself’: a qualitative study to explore the factors influencing adolescents’ consumption of sugar-sweetened beverages and sugary snacks from a socio-ecological perspective
Source: Public Health Nutr. 2022 May 18;25(9):2465–74. doi: 10.1017/S1368980022001185 (PMC9991719; doi:10.1017/S1368980022001185)
Supplement: Supplementary file 1 [file S1368980022001185sup.zip › S1368980022001185sup001.docx]

**Supplementary Table 1** Characteristics of the eight schools

| School no. | 1 | 2 | 3 | 4 | 5 | 6 | 7 | 8 |
| --- | --- | --- | --- | --- | --- | --- | --- | --- |
| Location | Xin Zhuang District, New Taipei City | Zhonghe District,  New Taipei City | Xizhou Township, Changhua County | Xizhou Township, Changhua County | Yuanlin City, Changhua County | Erlin Township, Changhua County | Shetou Township, Changhua County | Beidou Township, Changhua County |
| Classes (grades 7 to 9) | 65 classes | 34 classes | 12 classes | 11 classes | 49 classes | 3 classes | 30 classes | 29 classes |
| Total number of students | 1,799 | 895 | 321 | 263 | 1,352 | 75 | 813 | 821 |
| Rural/urban setting | Urban | Urban | Rural | Rural | Rural | Rural | Rural | Rural |
| School beverage vending machine(s) | n/a | n/a | n/a | 1 | n/a | n/a | n/a | 2 |
| School sugary snack vending machine(s) | n/a | n/a | n/a | 1 | n/a | n/a | n/a | 1 |
| School store(s) | 1 | 1 | 1 | n/a | 1 | n/a | n/a | n/a |
| Water dispensers | 61 | 38 | 6 | 8 | 29 | 7 | 13 | 28 |
| Food environment surrounding schools (less than 10-min walk) | 1 KFC; 1 McDonald's; 14 convenience stores; 5 supermarkets;  12 hand-shaken drink shops; 13 breakfast shops | 1 KFC; 1 McDonald's; 12 convenience stores; 6 supermarkets; 13 hand-shaken drink shops; 15 breakfast shops | 1 supermarket; 4 convenience stores; 6 hand-shaken drink shops; 13 breakfast shops | 2 grocery stores; 1 breakfast shop | 1 supermarket; 6 convenience stores; 4 hand-shaken drink shops; 4 breakfast shops | 1 convenience store; 2 hand-shaken drink shop | 1 supermarket; 4 convenience stores; 8 hand-shaken drink shops; 10 breakfast shops | 1 McDonald's; 1 KFC; 3 convenience stores; 9 hand-shaken drink shops; 2 breakfast shops |
| School food policy | n/a | n/a | Changhua County has banned SSBs on campus since 2017; however, this does not mean that all SSBs are forbidden at school. Stores and vending machines at school can sell fresh milk, 100% fruit juice, buttermilk and soy milk (fewer than 250 calories and less than 30% calories provided by added sugar), and other drinks with no sugar. In addition, SSBs carried in by students are not covered by the regulation. | | | | | |

**Supplementary** **Table 2** Interview guide for exploring factors influencing SSB and sugary snack consumption

| Topics and questions | Follow-up questions |
| --- | --- |
| I. Enquiry about current SSB and sugary snack consumption status |  |
| 1. Do you have a habit of drinking SSBs/eating sugary snacks every week? | 1. What type of SSBs/sugary snacks? 2. How often do you drink SSBs/eat sugary snacks every week? 3. When do you usually drink SSBs/eat sugary snacks? 4. How do you obtain SSBs/sugary snacks? (to explore whether participants buy them by themselves or receive them from their peers/parents/teachers) |
| II. Enquiry about individual factors influencing SSB and sugary snack consumption |  |
| 1. How much allowance do you use to buy SSBs/sugary snacks every week? | 2.a Where do you usually buy SSBs/sugary snacks? |
| 1. What do you think are the benefits of drinking SSBs/eating sugary snacks? | 3.a What do you think are the harmful effects of drinking SSBs/eating sugary snacks? |
| III．Enquiry about interpersonal factors influencing SSB and sugary snack consumption |  |
| 1. What factors or scenarios make you want to drink SSBs/eat sugary snacks? | 4.a How do you think your peers or friends influence your SSB and sugary snack consumption? Why?  4.b How do you think your parents influence  your SSB and sugary snack consumption? Why?  4.c How do you think your siblings influence  your SSB and sugary snack consumption? Why?  4.d How do you think your teachers influence  your SSB and sugary snack consumption? Why? |
| IV. Enquiry about environmental factors influencing SSB and sugary snack consumption |  |
| 1. Are there many shops selling SSBs/sugary snacks within a 15-min walk from your school? | 5.a How many convenience stores, hand-shaken drink shops, breakfast shops, street food vendors, and cake shops are around your school? |
| 1. How often are SSBs/sugary snacks present in your home? | 6.a What type of SSBs/sugary snacks are present? Who buys SSBs/sugary snacks? |
| V．Enquiry about societal factors influencing SSB and sugary snack consumption | |
| 1. Are you aware of any food policies regarding SSBs or sugary snacks implemented in your school? | 7.a If yes, what are the contents of these food policies?  7.b Do your schoolmates or classmates follow these rules? |
| 1. Are you aware of SSB or sugary snack advertisements in your life? | 8.a If yes, what are the contents of these food advertisements?  8.b Where do you see them? (TV, internet, social media, etc.)  8.c How do you think these food advertisements influence your SSB and sugary snack consumption? |

**Supplementary Table 3** Codes, categories, and themes generated from the semistructured interviews with junior school students ages 12-14

|  | Code | Rural/urban setting | Category | Themes |
| --- | --- | --- | --- | --- |
|  | Quench thirst | Both | Physiological factors | Intrapersonal factors influence SSB and sugary snack consumption |
|  | Reduce hunger |  |  |  |
|  | Reduce sleepiness |  |  |  |
|  | Feel bad/angry/stressed/bored/tired | Both | Psychological factors |  |
|  | Perceived motivation | Urban |  |  |
|  | Addicted to sugar | Both |  |  |
|  | Amount of allowance | Both | Individual economic factors |  |
|  | Prices of SSBs and sugary snacks |  |  |  |
|  | Tastes sweet/good | Both | Taste preferences |  |
|  | Tastes better than water |  |  |  |
|  | Parents' SSB and sugary snack consumption | Both | Negative parental influences | Interpersonal factors influence SSB and sugary snack consumption |
|  | Lack of parental supervision |  |  |  |
|  | Provided by parents |  |  |  |
|  | Parents as positive role models | Both | Positive parental influences |  |
|  | Parental supervision and control |  |  |  |
|  | Went with siblings to purchase | Both | Negative sibling influences |  |
|  | Food sharing behaviour between siblings |  |  |  |
|  | Perceived peers’ SSB and sugary snack consumption | Both | Negative peer influences |  |
|  | Food sharing behaviour among peers |  |  |  |
|  | Food recommendations from peers |  |  |  |
|  | Peer pressure |  |  |  |
|  | Perceived teachers' SSB and sugary snack consumption | Both | Negative teacher influences |  |
|  | Lack of supervision from teachers |  |  |  |
|  | Teachers use SSBs or sugary snacks to reward students |  |  |  |
|  | Teachers as positive role models | Both | Positive teacher influences |  |
|  | Teachers' supervision and control | Both |  |  |
|  | Teachers' punishments | Rural |  |  |
|  | Accessibility of SSBs and sugary snacks at home | Both | Food environment at home | SSB and sugary snack consumption are influenced by environmental context |
|  | Vending machines at schools | Rural | Food environment at school |  |
|  | Stores at schools | Both |  |  |
|  | Convenience of purchasing SSBs and sugary snacks in the neighbourhood near home | Both | Food environment in the neighbourhood near home |  |
|  | Inconvenience of purchasing SSBs and sugary snacks in the neighbourhood near home | Rural |  |  |
|  | Convenience of purchasing SSBs and sugary snacks in the neighbourhood near school | Both | Food environment in the neighbourhood near school |  |
|  | No specific SSB policy | Urban | School food policy |  |
|  | Some types of SSBs are prohibited | Rural |  |  |
|  | Breakfast culture | Both | Food culture | Societal-level characteristics influence SSB and sugary snack consumption |
|  | Hand shaken drink culture |  |  |  |
|  | Food advertising | Both | Food advertising |  |
